# Clinical characteristics, radiological features and outcomes in pulmonary involvement of cryoglobulinemia

**DOI:** 10.1186/s13023-024-03159-0

**Published:** 2024-05-02

**Authors:** Hong-xiao Han, Wei Su, Xinlun Tian, Dao-bin Zhou, Jian Li, Xin-xin Cao

**Affiliations:** 1 Department of Hematology, Peking Union Medical College Hospital, Chinese Academy of Medical Sciences and Peking Union Medical College, No.1 Shuaifuyuan, 100730 Beijing, China; 2 Department of Laboratory Medicine, Peking Union Medical College Hospital, Chinese Academy of Medical Sciences and Peking Union Medical College, Beijing, China; 3 Department of Pulmonary and Critical Care Medicine, Peking Union Medical College Hospital, Chinese Academy of Medical Sciences and Peking Union Medical College, Beijing, China; 4 State Key Laboratory of Complex Severe and Rare Diseases, Peking Union Medical College Hospital, Beijing, China

**Keywords:** Cryoglobulinemia, Diffuse ground-glass opacity, Alveolar hemorrhage, Rituximab

## Abstract

**Background:**

Cryoglobulinemia with pulmonary involvement is rare, and its characteristics, radiological findings, and outcomes are still poorly understood.

**Methods:**

Ten patients with pulmonary involvement of 491 cryoglobulinemia patients at Peking Union Medical College Hospital were enrolled in this retrospective study. We analyzed the characteristics, radiological features and management of pulmonary involvement patients, and compared with those of non-pulmonary involvement with cryoglobulinemia.

**Results:**

The 10 patients with pulmonary involvement (2 males; median age, 53 years) included three patients with type I cryoglobulinemia and seven patients with mixed cryoglobulinemia. All of 10 patients were IgM isotype cryoglobulinemia. All type I patients were secondary to B-cell non-Hodgkin lymphoma. Four mixed patients were essential, and the remaining patients were secondary to infections (*n* = 2) and systemic lupus erythematosus (*n* = 1), respectively. Six patients had additional affected organs, including skin (60%), kidney (50%), peripheral nerves (30%), joints (20%), and heart (20%). The pulmonary symptoms included dyspnea (50%), dry cough (30%), chest tightness (30%), and hemoptysis (10%). Chest computed tomography (CT) showed diffuse ground-glass opacity (80%), nodules (40%), pleural effusions (30%), and reticulation (20%). Two patients experienced life-threatening diffuse alveolar hemorrhage. Five patients received corticosteroid-based regimens, and four received rituximab-based regimens. All patients on rituximab-based regimens achieved clinical remission. The estimated two-year overall survival (OS) was 40%. Patients with pulmonary involvement had significantly worse OS and progression-free survival than non-pulmonary involvement patients of cryoglobulinemia (*P* < 0.0001).

**Conclusions:**

A diagnosis of pulmonary involvement should be highly suspected for patients with cryoglobulinemia and chest CT-indicated infiltrates without other explanations. Patients with pulmonary involvement had a poor prognosis. Rituximab-based treatment may improve the outcome.

## Introduction

Cryoglobulinemia is defined as the presence of cryoglobulins in the serum that precipitate in vitro at temperatures below 37 °C and dissolve with rewarming [1]. According to the composition of the immunoglobulin, three types of cryoglobulins can be classified [2]. Type I cryoglobulins consist of monoclonal immunoglobulins, and monoclonal gammopathy of undetermined significance is seen in ∼ 40% of patients with type I cryoglobulinemia, followed by multiple myeloma, Waldenström’s macroglobulinemia or small lymphocytic lymphoma/chronic lymphocytic leukemia (SLL/CLL) [3, 4]. Type II cryoglobulins are composed of monoclonal immunoglobulins with rheumatoid factor (RF) activity and polyclonal immunoglobulins, and type III cryoglobulins are characterized by polyclonal immunoglobulins. The latter two types are referred to as mixed cryoglobulins. Hepatitis C virus (HCV) infection is predominantly associated with mixed cryoglobulinemia [5]. Other common causes include additional infections, such as human immunodeficiency virus and hepatitis B virus; connective tissue diseases; and lymphoproliferative disorders. Nearly 10% of patients remain classified as essential [6]. 

Clinical presentations of cryoglobulinemia vary from asymptomatic and fatigue to heart failure and rapidly diffuse alveolar hemorrhage (DAH). The most commonly affected organ is the skin, which presents with purpura (75-90%), Raynaud’s phenomenon (20-30%), and distal ulcers (5-15%). The additional commonly involved systems include joints (50-80%), peripheral nerves (50-75%) and kidney (30-40%) [6–8]. Involvement of other major organs, such as the central nervous system (CNS), gastrointestinal (GI) tract, heart, and lung, is rare but associated with poor prognosis [9]. 

To date, few studies and case series of pulmonary involvement in cryoglobulinemia have been reported because of its rarity. Pulmonary involvement occurs in less than 5% of patients with cryoglobulinemia [3, 6, 7]. Pulmonary symptoms vary from dry cough and mild-to-moderate shortness of breath to acute DAH. Some studies [9, 10] have reported cryoglobulinemia with DAH as life-threatening cryoglobulinemia associated with high mortality. However, there are no studies focusing on patients with pulmonary involvement.

In this study, we described the clinical characteristics, imaging presentation, and management of cryoglobulinemia patients with pulmonary involvement. Furthermore, we compared patients of cryoglobulinemia with and without pulmonary involvement.

## Methods

### Study population

This retrospective study was conducted among patients with cryoglobulinemia at Peking Union Medical College Hospital between January 2015 and December 2022. The inclusion criteria for the study were: (i) detectable cryoglobulins in the serum; (ii) chest computed tomography (CT) findings such as diffuse ground-glass opacity (GGO), reticulation, diffuse nodules, multiple patches, pleural effusions, and DAH; and (iii) exclusion of other diagnoses with pulmonary lesions. Informed consent was obtained from all patients, and the protocol was approved by the Peking Union Medical College Hospital Ethics Committee. The study was performed in accordance with the ethical standard laid down in the 1964 Declaration of Helsinki and its later amendments.

### Cryoglobulin detection and laboratory evaluation

The process of testing for cryoglobulins was consistent with those in previous reports [11]. All peripheral blood samples were initially kept at 37 ℃ until serum separation. After warm clotting and centrifugation, the clear serum was preserved at 4 °C for seven days and observed for the formation of cryoprecipitates. The resulting precipitate was washed and rewarmed to 37 °C. All positive cryoglobulins were analyzed by immunoelectrophoresis to determine the type of cryoglobulin according to Brouet’s criteria [2]. Cryoglobulin quantification was analyzed using Wintrobe tubes revealing the cryocrit (packed volume of the precipitate relative to the original serum volume) and direct quantification of immunoglobulins in combination with agarose gel electrophoresis [12]. 

Laboratory evaluation included complete blood count, liver and kidney function tests, urinalysis, concentration of serum complement 3 (C3), complement 4 (C4) fraction, serum RF level, serum/urine immunofixation electrophoresis (IFE), serum protein electrophoresis (SPE) and 24-hour urine protein.

### Clinical presentation and underlying disease

Clinical data included demographic characteristics, clinical manifestations, coexisting medical conditions, chest CT scans and pulmonary function tests (PFTs). Symptoms related to cryoglobulinemia were defined as previous study [13]. The PFT data were interpreted according to the American Thoracic Society (ATS) and European Respiratory Society (ERS) criteria [14]. 

The underlying diseases included (i) hematological disease [15], including diffuse large B-cell lymphoma (DLBCL) and SLL/CLL, which were compatible with the 2016 version of the World Health Organization classification of neoplastic diseases; (ii) infections, including (a) HCV infection, positivity for anti-HCV antibodies and presence of HCV-RNA in serum by PCR [16], and (b) Epstein‒Barr virus (EBV) infection, presence of EBV-DNA in serum by PCR [17]; and iii) systematic lupus erythematosus (SLE), based on the European League Against Rheumatism (EULAR) 2019 criteria [18].

### Treatment and outcomes

Treatment was divided into the following categories: (i) corticosteroid-based treatment and (ii) rituximab-based treatment. The response to treatment was consistent with that reported by previous study [13]. Overall survival (OS) was defined as the time from diagnosis to the date of death or last follow-up. Progression-free survival (PFS) was defined as the time from the date of diagnosis to the date of underlying disease progression, cryoglobulinemia progression, death from any cause or last follow-up. The last follow-up was January 28, 2023.

### Statistical analysis

Clinical and imaging data were analyzed using descriptive statistics. Continuous variables are presented as the median and range, and categorical variables are presented as the number and percentages. The Mann‒Whitney U test was used for continuous variables, whereas Fisher’s exact test was used to compare categorical variables between two groups. Kaplan‒Meier analysis was applied for survival analysis, with survival curves being compared using the log-rank test. Statistical analysis was performed using SPSS 26.0 software (IBM Corp., Armonk, NY, USA), and the significance end point was set to *P* < 0.05.

## Results

### Patient characteristics

A total of 491 patients were diagnosed with cryoglobulinemia at Peking Union Medical College Hospital between January 2015 and December 2022. A total of 101 (20.1%) patients had type I cryoglobulinemia, 390 (79.4%) patients had mixed cryoglobulinemia, including 91 (18.5%) with type II, and 299 (60.1%) with type III. A total of 243 (49.5%) patients were asymptomatic, and the remaining patients (50.5%) had at least one organ involved. Overall, 10 (2.0%) patients (2 males and 8 females) fulfilled the inclusion criteria accounted for 2.0% of all cryoglobulinemia patients (*n* = 491). Patients with pulmonary involvement included 3 with type I cryoglobulinemia and 7 with mixed cryoglobulinemia, including 4 patients with type II and 3 patients with type III. For patients with pulmonary involvement, the median age at diagnosis was 53 years (range, 30–73 years), and the median duration from symptom onset to disease diagnosis was 8 months (range, 2–49 months).

We compared the baseline characteristics of type I cryoglobulinemia patients with (*n* = 3) and without pulmonary involvement (*n* = 98). We also compared the baseline characteristics of mixed cryoglobulinemia patients with (*n* = 7) and without pulmonary involvement (*n* = 383). The demographic and clinical characteristics are presented in Tables 1 and 2. Compared to type I patients without pulmonary involvement, patients with pulmonary involvement tended to have a higher RF titer (7.0 vs. 4.6 IU/ml, *P* = 0.065). Out of 390 mixed cryoglobulinemia patients, those with pulmonary involvement had higher frequencies of skin involvement (71.4% vs. 29.2%, *P* = 0.046), renal involvement (57.1% vs. 20.9%, *P* = 0.041), and peripheral nerve involvement (42.9% vs. 6.8%, *P* = 0.011) compared to those without pulmonary involvement. Furthermore, mixed cryoglobulinemia patients with pulmonary involvement showed a markedly higher RF titer than those without pulmonary involvement (226.1 vs. 98.0 IU/ml, *P* = 0.048).

**Table 1:** Clinical characteristics of type I cryoglobulinemia patients with or without pulmonary involvement

Clinical characteristics	All patients of type I cryoglobulinemia (*n* = 101)	Patients with pulmonary involvement(*n* = 3)	Patients without pulmonary involvement (*n* = 98)	P value
Male, n (%)	63 (62.4)	0 (0.0)	63 (64.3)	--
Age, years, median (range)	61 (20–81)	69 (52–73)	61 (20–81)	0.496
Cause, n (%)				
Essential, n (%)	6 (5.9)	0 (0.0)	6 (6.1)	--
Hematological disease, n (%)	90 (89.1)	3 (100.0)	87 (88.8)	1.000
Connective tissue disease, n (%)	3 (14.5)	0 (0.0)	3 (14.7)	--
Infectious disease, n (%)	2 (2.0)	0 (0.0)	2 (2.0)	--
Involved organ, median (range)	1 (0–5)	1 (1–4)	1 (0–5)	0.357
Skin lesion, n (%)	53 (52.5)	1 (33.3)	52 (53.1)	0.603
Renal involvement, n (%)	19 (18.8)	1 (33.3)	18 (18.4)	0.469
Peripheral nerve, n (%)	23 (22.8)	0 (0.0)	23 (23.5)	--
Joint involvement, n (%)	20 (19.8)	1 (33.3)	19 (19.4)	0.488
Cardiac involvement, n (%)	4 (4.0)	1 (33.3)	3 (3.1)	0.115
Positive of serum/urine immunofixation electrophoresis, n (%)	82 (81.2)	2 (66.7)	80 (81.6)	0.469
Isotype of cryoglobulin				
IgMκ, n (%)	52 (51.5)	2 (66.7)	50 (51.0)	1.000
IgMλ, n (%)	11 (10.9)	1 (33.3)	10 (10.2)	0.295
IgGκ, n (%)	21 (20.1)	0 (0.0)	21 (21.4)	--
IgGλ, n (%)	17 (16.8)	0 (0.0)	17 (17.3)	--
Concentration of cryoprecipitate, mg/L, mean (range)	169.8 (12.9-13046.5)	100.9 (24.3–3463.0)	171.1 (12.9-13046.5)	0.844
RF, IU/mL, median (range)	4.8 (0.3-11404.9)	7.0 (1.0-9.4)	4.6 (0.3-11404.9)	**0.065**
C3, g/L, median (range)	0.805 (0.158–1.839)	1.057 (0.721–1.142)	0.797 (0.158–1.839)	0.426
C4, g/L, median (range)	0.112 (0.001–0.486)	0.198 (0.050–0.225)	0.111 (0.001–0.486)	0.605

**Table 2:** Clinical characteristics of mixed cryoglobulinemia patients with or without pulmonary involvement

Clinical characteristics	All patients of mixed cryoglobulinemia (*n* = 390)	Patients with pulmonary involvement(*n* = 7)	Patients without pulmonary involvement (*n* = 383)	P value
Male, n (%)	140 (35.9)	2 (28.6)	138 (36.0)	0.289
Age, years, median (range)	53 (16–87)	55 (30–69)	53 (16–87)	0.415
Cause, n (%)				
Essential, n (%)	118 (30.3)	4 (57.1)	114 (29.8)	0.206
Hematological disease, n (%)	19 (4.9)	0 (0.0)	19 (5.0)	--
Connective tissue disease, n (%)	177 (45.4)	1 (14.3)	176 (46.0)	0.199
Infectious disease, n (%)	76 (19.5)	2 (28.6)	74 (19.3)	0.896
Involved organ, median (range)	1 (0–6)	3 (1–5)	1 (0–6)	0.221
Skin lesion, n (%)	117 (30.0)	5 (71.4)	112 (29.2)	**0.046**
Renal involvement, n (%)	84 (21.5)	4 (57.1)	80 (20.9)	**0.041**
Peripheral nerve, n (%)	29 (7.4)	3 (42.9)	26 (6.8)	**0.011**
Joint involvement, n (%)	29 (7.4)	2 (28.6)	27 (7.0)	0.154
Cardiac involvement, n (%)	7 (1.8)	1 (14.3)	6 (1.6)	0.282
Gastrointestinal tract, n (%)	6 (2.4)	0 (0.0)	6 (2.5)	--
Type II cryoglobulinemia, n (%)	91 (23.3)	4 (57.1)	87 (22.7)	0.055
IgMκ, n (%)	72 (18.5)	4 (57.1)	68 (18.0)	**0.024**
IgMλ, n (%)	12 (3.1)	0 (0.0)	12 (3.1)	--
IgGκ, n (%)	4 (1.0)	0 (0.0)	4 (1.0)	--
IgGλ, n (%)	1 (0.3)	0 (0.0)	1 (0.3)	--
IgAκ, n (%)	1 (0.3)	0 (0.0)	1 (0.3)	--
IgAλ, n (%)	1 (0.3)	0 (0.0)	1 (0.3)	--
Type III cryoglobulinemia, n (%)	299 (76.7)	3 (42.9)	296 (77.3)	0.055
Concentration of cryoprecipitate, mg/L, mean (range)	77.2 (10.5-47119.0.0)	91.0 (34.8–3463.0)	75.9 (10.5-47119.0)	0.332
RF, IU/mL, median (range)	65.0 (3.0-25100.0)	226.1 (3.0-19489.2)	98.0 (4.2-25100.0)	**0.048**
C3, g/L, median (range)	0.783 (0.044–2.054)	0.662 (0.283–1.121)	0.817 (0.044–2.054)	0.189
C4, g/L, median (range)	0.105 (0.001–1.020)	0.069 (0.002–0.194)	0.105 (0.001–1.020)	0.264

All patients with pulmonary involvement experienced persistent pulmonary symptoms, which included dyspnea (50%), dry cough (30%), chest tightness (30%), and hemoptysis (10%). The other most common clinical manifestations were purpura (50%) and proteinuria (50%), followed by renal function impairment (40%), hematuria (40%), sensory neuropathy (30%), arthralgia (20%), heart failure (20%), and ulcer (10%). The detailed clinical characteristics of patients with pulmonary involvement are shown in Table 3. Six patients had additional organs affected by cryoglobulinemia. Skin (*n* = 6) was the most commonly involved organ, followed by kidney (*n* = 5), peripheral nerves (*n* = 3), joints (*n* = 2), and heart (*n* = 2). Furthermore, both patients with cardiac involvement presented with heart failure. Echocardiography or cardiac magnetic resonance revealed decreased ejection fraction, and massive pericardial effusion in these patients. All type I patients were secondary to B-cell non-Hodgkin lymphoma, including DLBCL (*n* = 2) and SLL/CLL (*n* = 1). Among the 7 patients with mixed cryoglobulinemia, 4 patients were essential, and the remaining 3 patients were secondary to infections (*n* = 2), including HCV (*n* = 1) and EBV (*n* = 1), and SLE (*n* = 1).

**Table 3:** Demographics and clinical characteristics of cryoglobulinemia patients with pulmonary involvement

Patient	Sex, Age	Underlying Disease	Pulmonary Presentation	Other Involvement	Cryoglobulin Detection			Treatments and Outcomes
					Isotype	Cryocrit (n, %)	Cryoglobulin level, mg/L	
#1	Female 69 y	B-NHL	Dyspnea	No	Type I (IgMλ)	< 1.0%	100.9	R-CHOP: PR^a^, CR^b^, then relapse^c^, PFS 10 m Death from underlying disease progression
#2	Female 52 y	B-NHL	Dyspnea, hemoptysis	Skin, kidney, heart	Type I (IgMκ)	NA	3463.0	CS + RTX: PR^a^, NR^b^, then relapse^c^, PFS 10 m Death from underlying disease progression
#3	Female 73 y	B-NHL	Dyspnea	No	Type I (IgMκ)	< 1.0%	24.3	Treatment abandoning Death from underlying disease progression
#4	Male 69 y	No	Dry cough	Skin, kidney	Type II (IgMκ)	< 1.0%	91.0	CS: NR^a^ Death from infection
#5	Female 55 y	HCV infection	Dry cough	Skin, joint, kidney, heart	Type II (IgMκ)	3.0%	NA	Antiviral therapy + DRC: CR^a^, PR^b^ HCV-RNA undetected
#6	Female 68 y	No	Chest tightness	Skin, peripheral nerves, kidney	Type II (IgMκ)	5.0%	2926.6	DRC: PR^a^
#7	Female 47 y	No	Dyspnea	No	Type II (IgMκ)	< 1.0%	839.4	CS: PR^a^, then relapse^d^, PFS 7 m
#8	Male 64 y	EBV infection	Chest tightness	Skin, peripheral nerves, joint, kidney	Type III (polyIgM)	< 1.0%	34.8	CS: NR^a^ Death from infection
#9	Female 31 y	No	Chest tightness	Skin, peripheral nerves	Type III (polyIgM)	< 1.0%	45.5	CS + CTX: PR^a^, CR^b^, then relapse, PFS 5 m
#10	Female 30 y	SLE	Dyspnea, dry cough	No	Type III (polyIgM)	< 1.0%	85.6	CS + MMF: PR^a^, CR^b^

B-NHL = B-cell non-Hodgkin’s lymphomas; CR = complete response; CS = corticosteroids; CTX = cyclophosphamide; DRC = rituximab, cyclophosphamide and dexamethasone; HCV = hepatitis C virus; EBV = Epstein‒Barr virus; MMF = mycophenolate mofetil; NR = no response; PR = partial response; R-CHOP = rituximab, cyclophosphamide, vincristine, and prednisone; RTX = rituximab; SLE = systemic lupus erythematosus

^a^clinical remission; ^b^laboratory response, ^c^underlying disease progression, ^d^cryoglobulinemia progression

### Laboratory findings

The median concentration of cryocrit for patients with pulmonary involvement was 100.9 mg/L (range, 24.3–3463 mg/L), all of which was IgM isotype, including 6 with IgMκ, 1 with IgMλ, and 3 with poly IgM. Serum complement levels were evaluable in all patients with pulmonary involvement: seven patients had a decrease in C3 serum levels, and the median concentration was 0.489 g/L (range, 0.283–0.681 g/L), and five patients had a decrease in C4 serum levels, and the median concentration was 0.016 g/L (range, 0.002–0.076 g/L). Out of 9 patients with pulmonary involvement, 6 patients exhibited elevated titers of RF, and the median level of RF was 463.4 IU/ml (range, 41.5-19489.2 IU/ml).

In the 4 patients with renal function impairment, the median serum creatinine level was 176 µmol/L (range, 99–299 µmol/L). Five patients experienced proteinuria, with a median 24-hour urine protein level was 1.47 g/L (0.62–1.84 g/L), while four patients presented with hematuria. Additionally, serum IFE was positive in 3 patients (#2, #6, and #7) of 9 evaluable patients, all of which were IgMκ isotype, and urine IFE was positive in 2 patients (#1, #2) of 5 evaluable patients, including one light chain of κ and one light chain of λ. Furthermore, among 9 patients who underwent SPE, two patients (#6, #7) were positive, with M protein levels of 10.50 g/L and 21.60 g/L, respectively.

### Radiological presentation

**Fig. 1:**
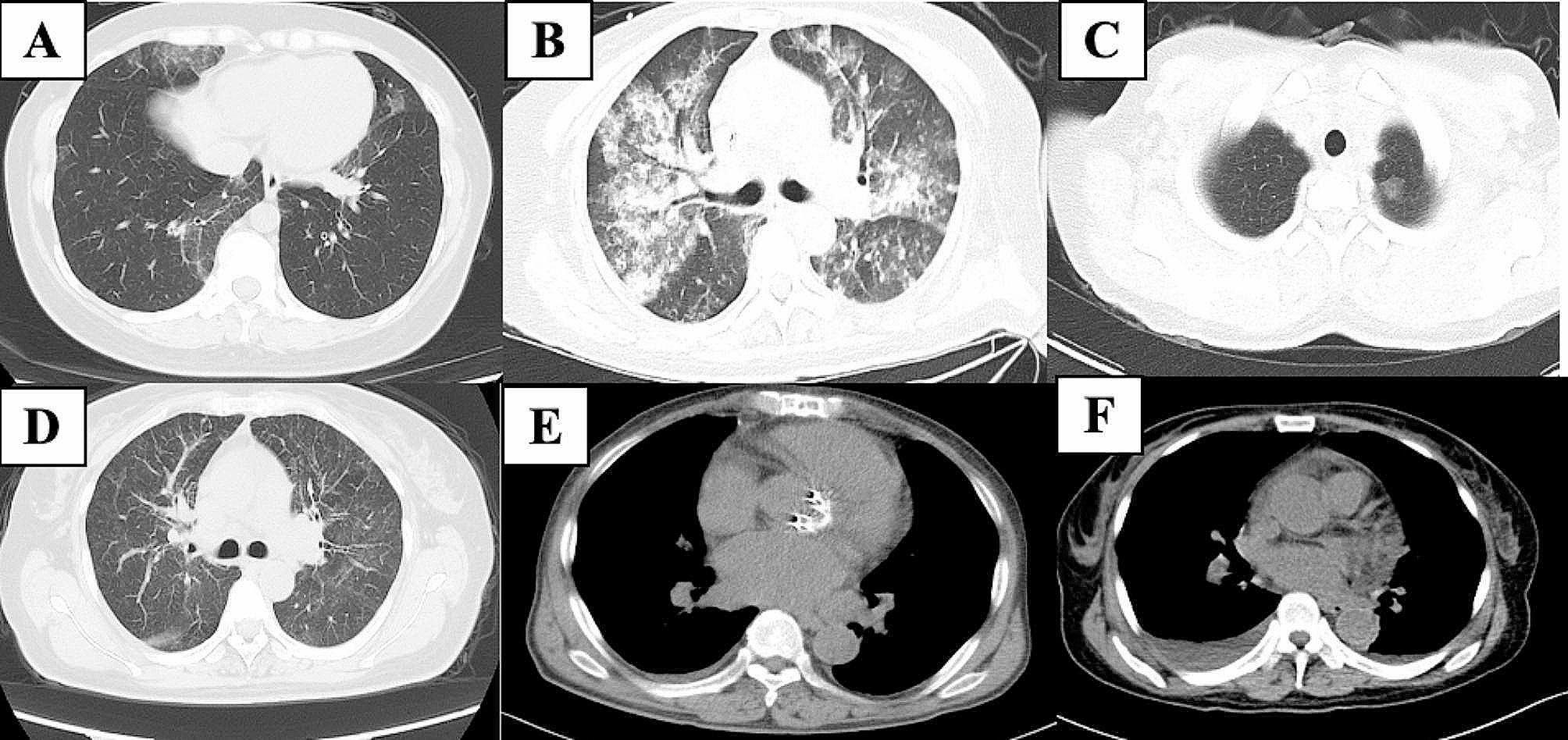
A-E, CT imaging findings associated with cryoglobulinemia and lung involvement. A, Diffuse, bilateral, patchy GGOs. B, Diffuse alveolar hemorrhage. C, Solitary large nodule in the right upper lobe. D, Diffuse, bilateral, multiple, small nodules. E, Bilateral pleural thickening. F, Bilateral pleural effusions

**Fig. 2:**
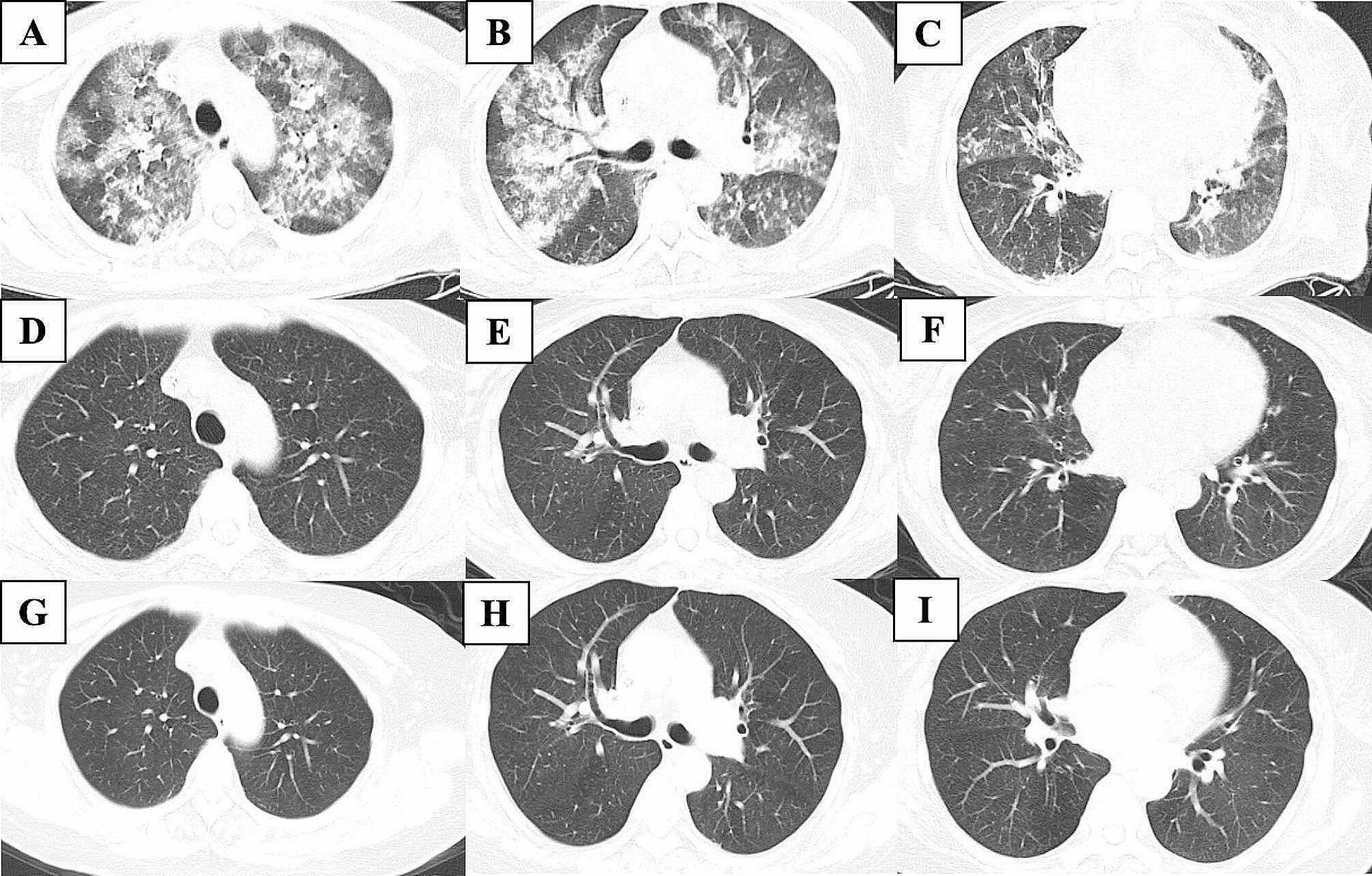
CT scans during the treatment of patient #2. A-C, Bilateral multiple patchy and diffuse GGOs along with symptoms of acute dyspnea and hemoptysis. D-F, Remarkable improvement of pulmonary infiltrates after high-dose corticosteroids and rituximab for 4 courses. G-I, Stability of symptoms and infiltrates on repeat chest CT 6 months after the episode of hemoptysis **(A)**

The radiological presentation of patients and examples of typical radiologic findings are shown in Table 4; Fig. 1. Both patients (#1, #2) with DAH showed diffuse bilateral GGO and multiple patches, and patient #2 also presented with pleural thickening and pleural effusions (Fig. 2A-C). Among the other 8 patients, 6 patients showed diffuse bilateral patchy GGOs, which were distributed mainly in the bilateral lower lobes of two patients (#5 and #10). Multiple, diffuse, small nodules were observed in four patients, two of whom (#8 and #9) had a large solitary nodule (maximum diameter > 10 mm) (Fig. 1C). Moreover, the CT scans revealed pleural thickening (50%), pleural effusions (30%), reticulations mainly in the bilateral lower lobes (20%), and bilateral interlobular septal thickening (10%).

**Table 4:** Radiological features and pulmonary function test results of pulmonary involvement in cryoglobulinemia

Patient	Diffuse bilateral patchy GGO	Nodules	Pleural thickening	Pleural effusions	Others	PFTs
#1	Yes	No	No	No	Reticulations	NA
#2	Yes	Yes	Yes	Yes	No	NA
#3	Yes	No	No	Yes	No	NA
#4	NA	Yes	Yes	No	Subpleural reticulations^a^	NA
#5	Yes (mainly in bilateral lower lobes)	No	No	Yes	Bilateral interlobular septa thickening^a^	NA
#6	Yes	No	Yes	No	No	FEV_1_/FVC 75.92%, TLC 79.4%, DLCO 42.3%
#7	Yes	No	No	No	No	FEV_1_/FVC 77.93%, TLC 100%, DLCO 72.9%
#8	No	Yes (mainly in right lobes)	Yes	No	No	NA
#9	Yes	Yes	No	No	No	NA
#10	Yes (mainly in bilateral lower lobes)	No	Yes	No	No	FEV_1_/FVC 89%, TLC 78.9%, DLCO 46.8%

DLCO = diffusion lung capacity for carbon monoxide; FEV1 = forced expiratory volume in 1 s; FVC = forced vital capacity; GGO = ground-glass opacity; TLC = total lung capacity

### Pulmonary function tests

PFTs were performed in three patients (#6, #7, and #10) (Table 4). The baseline PFTs of patient #6 revealed restrictive ventilatory dysfunction and diffusing-capacity defects. Likewise, patient #7 was subjected to baseline PFTs and demonstrated mild restrictive ventilatory dysfunction. Patient #10 showed mild restrictive ventilatory dysfunction with normal forced expiratory volume in 1 s (FEV_1_) and diffusing-capacity defects. After treatment with corticosteroids and mycophenolate mofetil (MMF), the predicted diffuse lung capacity for carbon monoxide (DLCO) value of patient #10 improved (from 46.8 to 67.2%) significantly along with normal restrictive ventilatory function.

### Treatment and outcomes

The treatments and outcomes of the 10 patients are listed in Table 3. One patient (#3) gave up treatment and died from underlying disease progression. Five (50%) patients received a corticosteroid-based regimen as first-line treatment, including corticosteroids alone (*n* = 3), corticosteroids and cyclophosphamide (CTX) (*n* = 1), and corticosteroids and MMF (*n* = 1). Four (40%) patients were treated with a rituximab-based regimen, including rituximab and corticosteriods (*n* = 1), R-CHOP (rituximab, cyclophosphamide, vincristine, and prednisone) (*n* = 1), DRC (rituximab, cyclophosphamide and dexamethasone) (*n* = 1), and antiviral agents and DRC (*n* = 1).

Of these 9 treated patients, 7 patients achieved clinical remission, consisting of one (#5) with complete remission (CR) and six with partial remission (PR). Furthermore, all 4 patients treated with rituximab-based regimens achieved clinical remission, while both patients (#4, #8) with no remission (NR) received corticosteroid treatment alone. Of the 5 patients evaluable for laboratory response, three patients (#1, #9 and #10) achieved CR, one (#5) achieved PR, and one (#2) showed NR. Moreover, patient #7 was treated with three lines of therapy and has been stable until now, which we previously reported [19]. 

**Fig. 3:**
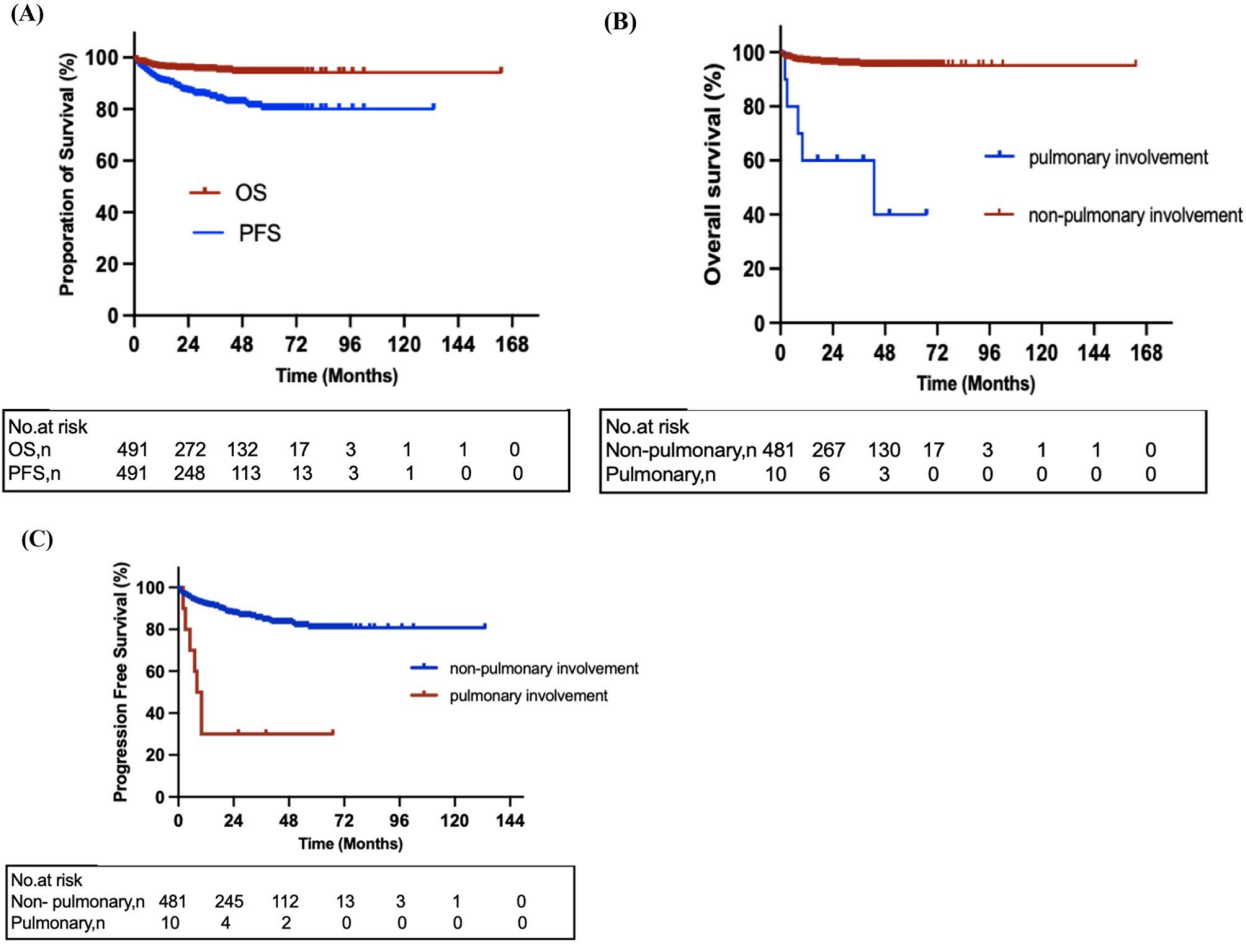
Comparison of survival among patients of cryoglobulinemia. (A) Overall survival (OS) and progression-free survival (PFS) of the whole cohort (*n* = 491). (B) OS of cryoglobulinemia patients with pulmonary involvement (*n* = 10) and those without pulmonary involvement (*n* = 481). (C) PFS of cryoglobulinemia patients with pulmonary involvement (*n* = 10) and those without pulmonary involvement (*n* = 481)

The median follow-up was 16 months (range, 2–67 months) for patients with pulmonary involvement. Five patients (50%) with pulmonary involvement died during follow-up. The causes of death included underlying disease progression (*n* = 3) and therapy-related infection (*n* = 2). The estimated three-year OS and PFS for all patients with cryoglobulinemia were 94.8% and 84.5%, respectively (Fig. 3A). Patients with pulmonary involvement had significantly worse OS and PFS than those without pulmonary involvement (two-year OS 40% vs. 95.6%, *P* < 0.0001; two-year PFS 30% vs. 87.7%, *P* < 0.0001; Fig. 3B, C).

## Discussion

Cryoglobulinemia with pulmonary involvement is extremely rare. To our knowledge, our study is the first reported cohort of cryoglobulinemia patients with pulmonary involvement compared with non-pulmonary involvement patients. This study provides new insights into the clinical characteristics, radiological findings, and management of cryoglobulinemia with pulmonary involvement.

Pulmonary involvement occurred in less than 5% of patients and seemed to be exceedingly rare in type I cryoglobulinemia [3, 6–8]. Trejo O et al. [6] reviewed 443 cryoglobulinemia patients and found 6 patients (1%) had pulmonary involvement with hemoptysis or pulmonary infiltrates on X-ray. According to our research, we found 10 patients diagnosed with pulmonary involvement accounted for 2.0% of all cryoglobulinemia patients. Patients with pulmonary involvement included 3 with type I cryoglobulinemia and 7 with mixed cryoglobulinemia. Most patients with pulmonary involvement were asymptomatic, dry cough and mild-to-moderate dyspnea. DAH was relatively uncommon, defined as life-threatening cryoglobulinemia and associated with extremely high mortality (up to 80-100%) [9, 20]. Consistent with previous studies, the pulmonary symptoms in our study included dyspnea (50%), dry cough (30%), chest tightness (30%), and hemoptysis (10%). Two patients presented with rapid DAH, and both died during follow-up. Compared to mixed cryoglobulinemia patients without pulmonary involvement, mixed patients with pulmonary involvement had higher frequencies of skin involvement, renal involvement, and peripheral nerve involvement. This could be due to that a significant proportion of mixed cryoglobulinemia patients (mainly type III patients) were asymptomatic.

The cryoglobulin in all patients with pulmonary involvement was IgM isotype. A previous study [10] showed that low C3 levels were associated with pulmonary involvement. However, comparing patients with pulmonary involvement and without pulmonary involvement, we found that patients with pulmonary involvement had a significantly higher RF titer, and no remarkable difference was found in C3 and C4 levels.

Few data are available on the radiographic presentation of cryoglobulinemia with pulmonary involvement. Generally, chest CT scans show various findings ranging from GGO to pulmonary fibrosis [21, 22]. An Italian study summarized 231 patients with mixed cryoglobulinemia [23]. Although mild dyspnea was observed in 26% of patients, only 2% (*n* = 4) of cases had abnormal chest CT scans. In another French study [24], pulmonary involvement consisted of intra-alveolar hemorrhage in four patients (1.7%) and bilateral interstitial infiltrates in one patient (0.4%) among 242 cases. In our cohort, bilateral and diffuse GGOs (1.7%) were the most common radiological findings, followed by pleural thickening (1.0%); diffuse, multiple, small nodules (1.0%); pleural effusions (0.6%); large solitary nodules (0.4%); reticulation (0.4%); and bilateral interlobular septa thickening (0.2%). Furthermore, the two patients with DAH presented with multiple patchy and diffuse GGOs.

Treatment should be designed according to the underlying diseases and the severity of clinical impairment. It is difficult to propose uniform management guidelines because of the heterogeneity in its manifestations and causations [25]. The Italian Study Group of Cryoglobulinemia (GISC) recommended rituximab as effective and safe for severe, not immediately life-threatening patients (LoE 1 A), and rituximab was more efficacious than conventional immunosuppressive treatments (1B) [26, 27]. All patients in our cohort received treatment initially, and the first-line treatment included corticosteroid-based regimens and rituximab-based regimens. Both patients with NR (#4, #8) received corticosteroid treatment alone, while all patients treated with rituximab-based regimens achieved good remission, which was consistent with GISC recommendations. Similar to previous findings [25, 28], the HCV-related patient in our study was treated with antiviral agents and DRC and achieved sustained virologic response and complete clinical remission. Furthermore, one essential patient did not achieve clinical remission by corticosteroid treatment alone but achieved clinical PR by DRC and clinical CR by BRD (bortezomib, rituximab, and dexamethasone) [19], which proved that bortezomib, given its direct effects on malignant plasma cells and antiangiogenic actions, is another promising biological agent.

Given the end-organ damage and life-threatening symptoms, patients with pulmonary involvement in cryoglobulinemia have poor survival. A French study of 242 cryoglobulinemia patients revealed that pulmonary involvement was an independent prognostic factor of poor outcomes [24]. In our cohort, patients with pulmonary involvement had an obviously poor prognosis compared with cryoglobuliemia patients without pulmonary involvement. Additionally, in our study, the two patients with DAH both died during follow-up because of underlying disease progression, consistent with previous studies showing that the mortality of rapid pulmonary hemorrhage in cryoglobulinemia patients was 80–100% [9, 10]. However, there were still no other survival data concerning the cohort of cryoglobulinemia patients with pulmonary involvement.

This study suffers from several limitations. First, this was a retrospective study and included a small number of cases. Patients with pulmonary involvement were only diagnosed with clinical pulmonary involvement in the absence of lung biopsy. However, all cases in our cohort were confirmed to have a cryoglobulinemia diagnosis and proved to have pulmonary abnormalities with no other explanation. Exploring the pathologic characteristics of cryoglobulinemia patients is needed in future studies. Second, we did not administer chest CT scans to all patients with cryoglobulinemia, and we may have missed some patients with moderate pulmonary involvement. Nevertheless, patients without chest CT scans did not have any pulmonary symptoms.

## Conclusions

Pulmonary involvement is rare in cryoglobulinemia. Pulmonary presentations vary from mild dyspnea to DAH, which is associated with high mortality. The chest CT scans mainly show diffuse GGOs. Patients of cryoglobulinemia with pulmonary involvement have a remarkably poor prognosis compared with non-pulmonary involvement patients with cryoglobulinemia. Rituximab-based treatment may be more effective.

## Data Availability

The datasets used and/or analysed during the current study are available from the corresponding author on reasonable request.

## Abbreviations

ATS: American Thoracic Society
B-NHL: B-cell non-Hodgkin’s lymphomas
BRD: Bortezomib, rituximab, and dexamethasone
C3: Complement 3
C4: Complement 4
CNS: Central nervous system
CR: Complete remission
CS: Corticosteroids
CT: Computed tomography
CTX: Cyclophosphamide
DAH: Diffuse alveolar hemorrhage
DLBCL: Diffuse large B-cell lymphoma
DLCO: Diffuse lung capacity for carbon monoxide
DRC: Rituximab, cyclophosphamide and dexamethasone
EBV: Epstein‒Barr virus
ERS: European Respiratory Society
EULAR: European League Against Rheumatism
FEV1: Forced expiratory volume in 1 s
FVC: Forced vital capacity
GGO: Ground-glass opacity
GI: Gastrointestinal
GISC: Italian Study Group of Cryoglobulinemia
HCV: Hepatitis C virus
IFE: Immunofixation electrophoresis
MMF: Mycophenolate mofetil
NR: No remission
OS: Overall survival
PFS: Progression-free survival
PFTs: Pulmonary function tests
PR: Partial remission
R-CHOP: Rituximab, cyclophosphamide, vincristine, and prednisone
RTX: Rituximab
SLE: Systematic lupus erythematosus
RF: Rheumatoid factor
SLL/CLL: Small lymphocytic lymphoma/chronic lymphocytic leukemia
SPE: Serum protein electrophoresis
TLC: Total lung capacity
